# Discontinuation of a randomised controlled trial in general practice due to unsuccessful patient recruitment

**DOI:** 10.3399/bjgpopen17X101085

**Published:** 2017-10-04

**Authors:** Wendelien H van der Gaag, Roxanne van den Berg, Bart W Koes, Arthur M Bohnen, Lonny MG Hazen, Wilco C Peul, Leen Voogt, Arianne P Verhagen, Sita MA Bierma-Zeinstra, Pim AJ Luijsterburg

**Affiliations:** 1 GP trainee and PhD student, Department of General Practice, Erasmus MC, University Medical Center, Rotterdam, The Netherlands; 2 GP trainee and PhD student, Department of General Practice, Erasmus MC, University Medical Center, Rotterdam, The Netherlands; 3 Professor of General Practice, Department of General Practice, Erasmus MC, University Medical Center, Rotterdam, The Netherlands; 4 GP, Department of General Practice, Erasmus MC, University Medical Center, Rotterdam, The Netherlands; 5 GP, Department of General Practice, Erasmus MC, University Medical Center, Rotterdam, The Netherlands; 6 Professor of Neurosurgery, Department of Neurosurgery, Leiden University Medical Center, Leiden, The Netherlands; 7 Patient and Board Member of 'De Wervelkolom', Dutch Association for Back Pain Patients, Lichtenvoorde, The Netherlands; 8 Associate Professor, Department of General Practice, Erasmus MC, University Medical Center, Rotterdam, The Netherlands; 9 Professor of Osteoarthritis and Related Disorders, Departments of General Practice and Orthopedics, Erasmus MC, University Medical Center, Rotterdam, The Netherlands; 10 Assistant Professor, Department of General Practice, Erasmus MC, University Medical Center, Rotterdam, The Netherlands

**Keywords:** primary health care, general practice, randomized controlled trial, patient recruitment, early termination of clinical trials, study design

## Abstract

**Background:**

A randomised controlled trial (RCT) in general practice, recruiting incident patients with (sub)acute sciatica, was discontinued because of insufficient recruitment.

**Aim:**

To describe factors that influenced the recruitment process and ultimately led to discontinuation of this trial, and to enable others to learn from this experience.

**Design & setting:**

A pragmatic RCT was designed to compare two pain medication prescription strategies for treatment of (sub)acute sciatica in general practice. After 1 year of patient recruitment, the trial was prematurely terminated.

**Method:**

To analyse the underperforming recruitment, patient information systems of 20 general practices were screened twice a month to search for eligible patients and identify reasons for non-eligibility. Secondly, after study termination, an open question was distributed to the participating GPs for their views on the recruitment process.

**Results:**

A total of 116 GPs from 37 general practices collaborated in the trial. Only eight of 234 patients were included after 12 months. The 22 GPs who offered their opinion on the main reasons for unsuccessful recruitment considered that these were the low incidence rate and strict eligibility criteria, a strong patient and/or GP preference, and time constraints.

**Conclusion:**

For this RCT, multiple factors were related to recruitment problems but it remains unknown which determinants prevailed. As the research question is unanswered but remains relevant, it is recommended that GPs’ daily practice is taken into account when designing an RCT, a pilot study should be performed for feasibility of recruitment, and GP assistants should be involved at an early stage.

## How this fits in

Research in the field of general practice is essential to expand evidence for guidelines in the diagnosis and treatment of diseases within primary care. However, in practice, study protocols are not always congruent with the reality in general practice. This article describes the rationale behind a randomised controlled trial that was prematurely discontinued due to insufficient patient recruitment. The following recommendations for future research are to design convenient protocols that facilitate referral to research as a routine task for GPs, with the least possible interference with daily regular practice, to perform a pilot study, and to outsource eligibility screening to trained GP assistants or dedicated research nurses.

## Introduction

General practice has changed rapidly in recent decades. Ageing populations and increasing care for patients with chronic diseases and (complex) multimorbidity demand expanding service profiles.^1^ These changing health needs have shaped the developments in general practice in the Netherlands and elsewhere. For example, both patient list size per GP and the average patient contact rate with the general practice have increased.^2–4^ Dutch GPs spend (on average) more time on non-patient bound activities (such as administration, management, and continuing vocational training) than their European counterparts.^1,5^ Task delegation to practice assistants or nurses is inevitable and has become regular practice. The question then arises whether conducting clinical research is more difficult in today’s general practices, due to changes in priority setting; that is, changes that might now be inevitable to keep the workload manageable.

For evidence-based decision making in general practice, clinical research is essential and most GPs are aware of this.^6,7^ Researchers, on the other hand, have to translate their research questions and ideas into concrete trial designs that are feasible in general practice. The design needs methodological soundness and adequate power to answer the research questions.^8,9^ A complicating factor is that the protocol must have minor interference with daily practice to be feasible.^10^ Thus, a delicate balance is required between patients and GPs’ needs, and research aims that will ultimately benefit the patient.

The authors developed and received funding for a pragmatic design of an RCT on sciatica, the most prevalent specific type of low back disorders seen by GPs. Current treatment of sciatica in general practice aims to enable patients to stay active and return to daily activities as soon as possible.^11^ Therefore, pain relief through adequate analgesic(s) prescription is an important condition; however, the best approach for prescription of pain medication in sciatica remains unclear.^12^ This RCT, the STEP-UP trial, aimed to assess the clinical and cost-effectiveness of immediate opioid pain medication (followed by step-down) versus stepped-up pain medication according to the current clinical GP guidelines, in patients with (sub)acute sciatica in general practice over a period of 12 weeks.^13^

During 12 months of recruiting in general practices, 23 patients were assessed for eligibility by the research team, of which only eight (3.4% of the target 234 patients) could be included in the trial. Because the inclusion rate was too slow to recruit a sufficient number of patients in the remaining time available, the study was prematurely terminated after 1 year.

The aim of this study was to describe determinants in the recruitment process that ultimately led to discontinuation of this trial.

## Method

### Trial design and setting

**Figure 1.**
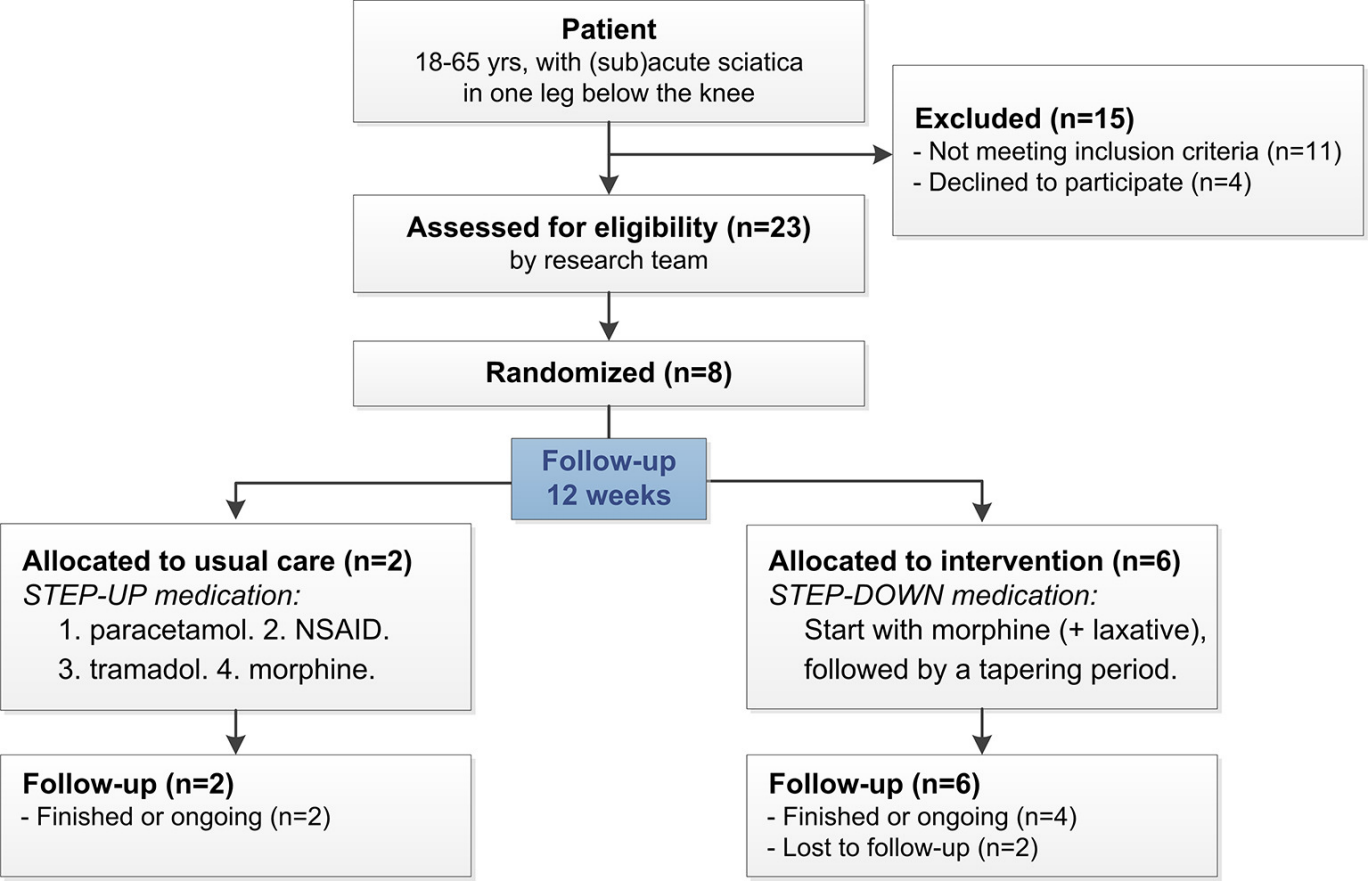
Flow diagram of the STEP-UP trial (pragmatic design RCT).

The STEP-UP trial was a pragmatic, multicentre, open-label, superiority RCT with a parallel group design, in general practices, with a 3-month follow-up, as shown in Box 1 and Figure 1. ^13^ Patients consulting their GP with (sub)acute sciatica were randomly allocated to receive stepped-up medication (step 1: paracetamol; step 2: non-steroidal anti-inflammatory drug [NSAID]; step 3: tramadol; step 4: morphine) or receive immediate morphine; followed by a taper period (step-down).

**Box 1.** STEP-UP trial design

Design	A pragmatic, multicentre, open-label, randomised controlled trial with parallel group design, in general practices with a 3-month follow-up period
Setting	General practices in the southwestern area of the Netherlands
Objective	To assess the clinical and cost-effectiveness of two pain medication prescription strategies in general practice over a 12-week period
Eligibility criteria	Inclusion criteria Age 18–65 years No use of opioids Radiating (pain) complaints in one leg below the knee Severity of radiating leg pain scored ≥7 on an 11-point numerical rating scale (0 = ‘no pain’; 10 = ‘worst pain imaginable’) Duration of the (pain) complaints <12 weeks Presence of at least one of the following symptoms: More pain on coughing, sneezing, or straining Decreased muscle strength in the leg Sensory deficits in the leg Decreased reflex activity in the leg Positive straight leg raising test
	Exclusion criteria An episode of radiating (pain) complaints in the preceding 6 months Back surgery in the past 3 years Treated with epidural injections Pregnancy Comorbidity that primary determines overall wellbeing (for example, an osteoporotic fracture, malignity, herpes zoster, or Lyme’s disease) Hypersensitivity to paracetamol, NSAID, or opioids Previous or active peptic ulcer Direct indication for surgery (fast progression of paresis of cauda equine syndrome) History of substance addiction or abuse
Outcome	Primary outcome was the severity of radiating leg pain measured daily over a 6-week follow-up period using an 11-point numerical rating scale (score range 0–10), with a higher score indicating more pain.

### Selection and recruitment of GP practices

It was calculated that the cooperation of at least 91 GPs was needed to recruit 234 patients with (sub)acute sciatica within 21 months.^14^ GP practices were identified in the southwestern area of the Netherlands —﻿ selected by zip code —﻿ and recruited by mail, followed by a phone call to make an appointment (practice visit or by phone with the choice up to GP) to explain more about the trial. The search area was expanded every month until the numbers were reached; and GP recruitment was continued when it was discovered that patient recruitment from the 91 GPs was behind schedule. A monthly newsletter was sent to update the GPs about the trial, and on request an e-mail reminder was sent every 2 weeks, containing information on eligibility criteria, how to invite patients to join and the consent procedure. Furthermore most general practices were visited regularly to remind GPs and practice nurses about the trial.

### Patient recruitment process

Patients were identified in two ways:

1. during GP consultations (1 April 201–30 March 2016), and
2. screening of GP information systems (29 September 2015–4 January 2016).

#### During the consultations

The GP performed physical examination, briefly informed eligible patients about the trial, invited them to participate and then notified the research team. The researcher contacted the patient to further assess eligibility, explain the informed consent procedure, and elaborate on any questions. Patients could consider participation in the study for as long as they wished, unless they were not using stronger medication than NSAIDs. After enrolment and randomisation by the research team, both patients and GPs were informed promptly by phone: the GP prescribed medication accordingly and patients could collect the medication the same day. Using this strategy, by September 2015 (after 6 months of recruiting) only one patient was included in the trial.

#### Screening of GP information systems

The GP information systems of 20 of the participating general practices (a total of 72 GPs) were screened systematically every 2 weeks during 3.5 months for patients that visited the general practice with International Classification of Primary Care (ICPC) code L86 (sciatica). The GP practices decided for themselves if they would participate in this screening option. If so, every 2 weeks they could assess the patients on the list for eligibility and send letters to invite them for study participation. After a week a reminder letter was sent. Both letters contained the patient information form, a response form, and a stamped return envelope. If patients returned the response form, they were called by the research team to further inform them about the trial, re-assess eligibility and for the informed consent procedure. After enrolment and randomisation by the research team, both patients and GPs were informed promptly by phone: the GP prescribed medication accordingly and patients could collect the medication the same day.

### Delayed inclusion and discontinuation of the study

When inclusion was clearly delayed, the problems were discussed during a GP network meeting in September 2015, with about 11 representatives of the participating general practices. Ten GPs were interviewed at their workplace and a research team meeting was scheduled to discuss the main reasons for delay and possible steps to be taken. A STEP-UP month was organised, where all participating GPs and practice nurses were visited to motivate them, strengthen their involvement, and enhance recruitment. A weekly newsletter was sent out to activate GPs and there was a voucher reward for every new patient admitted by a general practice.

Nevertheless, the study was terminated as the measures taken showed insufficient effect. After discontinuation (30 March 2016), each participating GP was sent a written survey, asking: *Why do you think we were unable to include a sufficient number of patients in the STEP-UP trial?*

## Results

### GP recruitment

GP recruitment started in January 2015 and patient recruitment in April 2015 with the cooperation of eight general practices and 36 GPs. The research question was considered relevant by the GPs that wanted to participate. For the negative responders, the main reasons for declining participation were lack of time, participation in other trials, or lack of interest in the topic. When patient recruitment was discontinued in March 2016, more than 450 general practices and GPs had been invited to join the trial; of these, 37 general practices with 116 GPs were participating in the study.

### Patient recruitment

**Figure 2.**
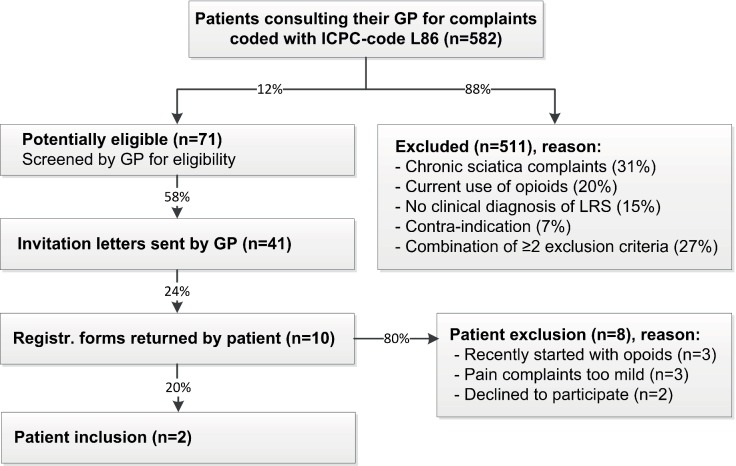
Systematic screening of GP information systems during a 3.5-month period. LRS = lumbosacral radicular syndrome.

Figure 2 shows the results of the systematic screening of the GP information systems in 20 general practices. Of the 582 patients screened during the 3.5-month time frame, 71 (12%) were potentially eligible. GPs sent invitations to 41 of these patients (58%) of whom 10 (24%) returned the registration form. Finally, of these, two patients were included and eight were either excluded or declined to participate.

### Reasons for insufficient recruitment

Following the project group meetings and interviews with GPs, factors that could have contributed to the low inclusion were identified.

1. *Strict eligibility criteria:* based on data from an earlier completed RCT (135 patients with [sub]acute sciatica recruited from 61 GPs over 18 months; May 2003–November 2004),^14^ an estimation was made of the number of GPs needed to recruit 234 patients within 21 months. However, for the present trial the earlier inclusion/exclusion criteria were adapted (that is, they were stricter). For example, in this study, a pain score (visual analogue scale) of ≥7 instead of ≥4 was required to participate, and criteria to prevent adverse events or abuse were added because this study involved NSAIDs and morphine. The effect of these adaptations on recruitment was greater than expected. In retrospect, a more conservative estimation of the speed of recruitment was needed.
2. *Incident case study:* the trial was designed to enrol patients consulting their GP with (sub)acute sciatica. Inclusion estimates were based on the incidence of sciatica in the Netherlands, which is estimated at 5 per 1000 persons/year in general practice.^15^ In addition, the incidence (all ages) of ICPC code L86 (sciatica) in general practice was 15.2 per 1000 patients/year in 2014, according to the NIVEL Primary Care Database.^16^ However, in fact, due to the restrictive eligibility criteria, only 12% of the patients with ICPC code L86 were eligible for participation, which results in approximately four eligible patients/year per full-time GP.

Of the 116 GPs, 22 (19%) responded to the written survey (that is one question about the reasons for recruitment failure) sent to each GP after discontinuation of the study. Their answers are presented in Box 2: 11 (50%) mentioned a strong patient preference and/or GP preference for either the step-up or step-down approach; six GPs (27%) mentioned the low incidence rate; and six (27%) the strict eligibility criteria. Other constraints included time and priority constraints, forgetfulness, and prevention of (further) treatment delay for their patients. A summary of reasons is shown in Table 1.

**Table 1.** Summary of responses of 22/116 (19%) GPs

Reasons for insufficient recruitment	GPs: *n* (%)^a^
Shared decision-making process: (strong) patient preference and/or GP preference for specific medication, diagnostics, randomisation outcome	11 (50)
Low incidence rate	6 (27)
Restrictive eligibility criteria	6 (27)
Priority setting: too many other responsibilities/administrative burden, time consuming/time pressure	6 (27)
No further delay wished	5 (23)
Forgetfulness	5 (23)
Lack of motivation	4 (18)
Other reasons	4 (18)

*^a^More than one answer was possible.*

**Box 2.** Responses of 22/116 (19%) GPs suggested reasons for insufficient recruitment

Number	Written responses of the 22 GPs	Summary of reasons
1	Lack of patients who fulfilled eligibility criteria. Most patients that considered participation didn’t want to use morphine. A few patients with a language barrier or contra-indication for the use of either morphine or NSAIDs. If, after this, patients could still be included, it was hard to include them and was too demanding in time and effort from the GP. As a GP I am already used to prescribe morphine in case of severe pain. In the trial design phase these problems were mentioned by GPs; for example in the GP network meeting, but I have the feeling this wasn’t taken into account enough.	Eligibility criteria Shared decision making Priority setting Other
2	The threshold for patients (and doctor) is (too) high when it comes to prescribing morphine for pain relief. It takes a lot of effort from us as GPs to use diagnostics and titrate patients to morphine: usually this takes 1–2 patient contacts a week. A referral to the neurologist is faster. The low response is a pity: in my opinion it is a very relevant research question.	Shared decision making Priority setting
3	My suspicion is that the researchers overestimated the incidence from ICPC L86. Maybe GPs encode inaccurately. Speaking for myself: when in doubt, if I can choose between L02, L03 or L86, I prefer L03 or L02. L86 seems to have much more consequences; as a GP I prefer some supporting diagnostics to code L86 with certainty. I consider the physical examination alone too limited to do so.	Incidence
4	It is hard to remember the trial at the right moment. What would help? A financial reward for the GP for admitting a patient to the trial. It’s a pity, but that is the case. With €50 per patient you would see an increase in trial participants.	Forgetfulness Motivation
5	Low amount of eligible patients. Because of this, I don’t think about including a patient in the trial at the right moment; I forget it.	Eligibility criteria Forgetfulness
6	When the trial was presented, we thought the research question to be very relevant. From the start of the trial I realised I found it hard to leave the choice for pain medication out of hands. In two cases with approximately the same amount of pain, you still choose a different pain management method per patient, depending on many other factors than just the pain. I didn’t want ‘the randomisation’ to decide this. But it’s a pity that this means we will not have an answer to the research question.	Shared decision making
7	It is difficult to ask a patient to participate in research before starting an intervention (for example, start of medication). So in this case it also didn’t help to select patients later with the screening, most of them were already using medication. And for ... research: the GP ‘just’ forgets about it. The GP assistant is the first contact moment with the patient; it would already help if they ... put a note in the patient's file about the possibility of participating in the trial.	Shared decision making Delay Forgetfulness
8	Too many research projects at the same time!	Motivation Priority setting
9	Low incidence of the disease. In 12 months I didn’t see any new patient with sciatica. Not enough time in daily practice for informed consent. For screening the patient information system for prevalent cases there is no binding contract concerning patient privacy for the research assistant.	Incidence Priority setting Other
10	Research topic not that interesting. Opioid pain medication often has a lot of side effects that are often underestimated. They made a phrase for this for a reason: opioid rotation. Well, and then a lot of patients die after a little while. Low frequency of patients with sciatica. Too many research projects.	Incidence Shared decision making Motivation Priority setting
11	Probably less ‘feeling’ with the research question. I had the experience that a patient I submitted was excluded for the trial. Patients often didn’t want to participate. Later in the trial I lost my motivation.	Shared decision making Motivation
12	The pain score threshold for inclusion in the trial was very high (inherent to the possibility of opioid medication as painkillers): that is just quite rare. And secondly: in such pain, patients have often already tried a lot of pain medication themselves, which makes participation in a trial and randomisation to the control arm feel like starting over with something you already knew it didn’t help.	Shared decision making Eligibility criteria
13	The trial concerns a group of patients that demands and needs direct help. The research setting then causes an unnecessary and unwelcome delay.	Shared decision making Delay
14	Incidence of the disease in general practice is too low.	Incidence
15	Forgetfulness about the study before inclusion. Our general practice joined the trial in January 2016 only. The inclusion criteria were very specific and narrow; especially concerning the pain score.	Eligibility criteria Forgetfulness Other
16	Sciatica is apparently less prevalent than expected. Or maybe patients go to the physiotherapist by themselves and they don’t visit their GP anymore for this complaint? [Note: since 2005 a GP referral is no longer needed to consult a physiotherapist; everyone in the Netherlands is allowed direct access]	Incidence Other
17	Very limited number of patients that presented themselves for consultation with sciatica complaints; and then additionally were also eligible for participation in the trial.	Incidence Eligibility criteria
18	Attention in our general practice is mostly claimed by countless organisational tasks and duties like accreditation, new legislations (recently two new laws) and so on. We can’t handle more than sporadic and ad hoc duties.	Priority setting
19	These are often patients with impatience, demanding a quick solution. As a GP you want to take away the agitation (and sometimes the 'demanding' behaviour) and quickly offer a bit of relief and structure through explanation and medication. The procedure through trial participation is too exhaustive. As a GP, I don’t want 'this' patient to be randomised in the control arm; because I already changed my prescribing patterns a few years ago, so the treatment arm is how I currently treat my patients.	Shared decision making Delay
20	In the acute phase you already act on your findings; so if morphine is needed, you have already prescribed this before the trial comes into focus.	Delay
21	This is a proactive study, therefore I have to remember it at the moment itself. Usually the treatment of choice is the result of a —﻿ sort of —﻿ negotiation between patient and GP. Often patients already used paracetamol, or react like: 'Paracetamol?', and they feel unheard.	Shared decision making Forgetfulness
22	Myths about opioids by patients. Patients want to start with medication directly. In our general practice there’s often a language barrier which increases difficulty when it comes to explaining the trial and answering questionnaires.	Shared decision making Eligibility criteria Delay

In (unstructured) interviews GPs mentioned a few trends in general practice that also may have influenced the course of this trial:

1. *Changes in patient populations and prescription habits:* epidemiological research shows a tendency of increased production and utilisation of prescription opioid analgesics for the treatment of non-cancer pain in industrialised countries; this might also apply to our targeted study population.^17–21^ Furthermore, with increasing comorbidity and an ageing population, the use of NSAIDs and/or tramadol is contraindicated more often, which could accelerate the process from step 1 to 4, by skipping steps 2/3.
2. *Process of shared decision making and patient-centred care:* the decision-making process in modern general practice has become more patient-centred. The doctor–patient relationship is increasingly based on shared decision making, with respect for patients’ autonomy, instead of paternalism. The chosen approach largely depends on patient characteristics and their preference and expectations;^22,23^ for example, for pain medication, personal preferences can be strong. Some GPs mentioned that patients prefer drugs other than morphine, because morphine is considered to be too strong (for example, for fear of adverse events, or wanting to keep driving). Other patients, in contrast, request stronger pain medication and/or diagnostics. Thus, their GPs already have to negotiate to prevent unnecessary diagnostics. Consequently, patients on both sides of the spectrum did not want to be randomised.
3. *Priority setting:* the changes in both healthcare system and healthcare include societal developments, the transition of several disease treatments and general psychological care from secondary to primary care, decentralisation of youth and elderly care, implementation of new laws, and organising safe digital working systems; GPs spend a considerable amount of time on managing these processes. This may have (indirectly) influenced the present research process; both the willingness and/or time available to participate in research may decrease subsequently.

## Discussion

### Summary

This study described the rationale behind a prematurely discontinued RCT and identified several barriers that could have contributed to the low inclusion rate. The main study-related factors were the strict eligibility criteria and the trial design, aiming to *enrol* incident cases with an acute condition. The main external factors were societal developments and changes in patient populations, prescription habits, and the doctor–patient relationship. Research is needed to expand evidence for guidelines in the diagnosis and treatment of diseases within primary care. This article describes a trial that was not successful, to enable other researchers and GPs to learn from this experience. 

### Strengths and limitations

This study's main limitation was that the protocol seemed too complex to fit well into the daily clinic, for example resulting in a problem for patients with an acute need for pain medication due to the informed consent procedure. It was not possible to determine further strengths and limitations as the trial was prematurely discontinued.

### Comparison with existing literature

This experience is not new: achieving sufficient patient recruitment in clinical trials in general practice is an ongoing problem.^24–26^ Many prospective studies depend on GPs to recruit patients. However, for various understandable reasons, there is a discrepancy between the number of GPs that agree to participate and the number that actually recruit patients, even though they thought it was feasible in practice.^27,28^ This is why training of clinical staff —﻿ such as GPs, GP assistants, research nurses —﻿ is vital to improve patient recruitment, as mentioned by Donovan *et al*.^29,30^ Their research shows increases in trial participants after staff training programmes and individual tailored feedback and support. Bower *et al* suggests to increase public and professional engagement with research to improve participation by both clinicians and patients.^31^

Literature research in this field suggests that, in general, the recruitment rate (even in successful trials) is lower than anticipated.^32^ Apparently researchers are often too optimistic about the number of eligible patients, and the GPs’ time and ability to recruit patients.^33^ According to Lasagna’s Law, they overestimate the incidence of patient availability.^24^ This addresses the difficulty of predicting numbers by researchers, especially in studies with complex eligibility criteria and in studies that require incident cases of acute conditions.^34^ For future research, a pilot study should be performed (if financially feasible) in which the inclusion/exclusion criteria are tested before going full scale.^24,35^ Alternatively, as suggested by Haidich *et al*, patient enrolment patterns during the first 2 months can be analysed and will often predict the eventual ability of a trial to attain its target sample size.^36^

Some determinants are associated with a higher risk of recruitment failure; for example, restrictive eligibility criteria which is often a result of the difficult balance between diminishing risks for individual patients and maintaining the generalisability of trial results, and studies interfering with the GP’s decision-making process; that is, when recruiting patients has a direct impact on usual care after the randomisation process.^33^ A systematic review highlights the same main barriers for clinicians and patients experienced in the current study, including time constraints, potential impact on the doctor–patient relationship, concern for the patient, loss of professional autonomy, patient preferences, and/or difficulty with the consent procedure.^37^ Treweek *et al* suggests that a financial incentive for patients could have been a promising strategy to increase patient recruitment, next to an open trial design and the telephone reminders already used in this trial.^38^ The PRECIS wheel is a tool developed by Thorpe *et al* that can be used by researchers to help them designing a trial that is consistent with their purpose.^39^ Reflecting on this, because of the nature of the disease (sciatica) and the strength of the medication this trial was testing (morphine), the in- and exclusion criteria of the current trial were rather narrow, while the trial design itself was pragmatic. Comorbidities that were sometimes common, where reasons for exclusion in this trial. From that perspective, this trial appeared to be less pragmatic than orginally aimed for.

### Implications for research and practice

From the GPs’ viewpoint there was a contradiction between practising research or practicing medicine in the consultation room. As researchers, we should aim to close this gap by designing simple and convenient protocols that facilitate participation in research during busy consultation schedules. Training and support should be offered when needed.^40^ If possible, researchers should prevent too much interference in regular practice and create a format that fits in the daily routine tasks of GPs. Referring a patient to research should be as easy as referring someone to a specialist or paramedical discipline: preferably, one ‘click’ away, no paperwork involved, and using existing digital referral systems.

The introduction of practice nurses for both somatic and psychological care paved the way for task delegation in chronic care and psychosocial support. Regarding evidence-based medicine in primary care, it is worthwhile to investigate the possibilities of involving research nurses in general practice to make research a part of daily practice. However, the applicability of dedicated research nurses in general practice might be limited, both for logistic reasons (wide variation in staffing models worldwide), financial (who pays) and practical reasons (for example, access to patient information might be limited for research nurses because of privacy laws). Aiming for more involvement of GP assistants in the recruitment process is a more pragmatic and achievable solution. Compensation for the practice for the additional time and expenses related to the research in that case will be a point of discussion. Another possibility is to combine care and research into an integrated service/research clinic, as is more often seen in the UK.^31,41,42^

In summary, several recommendations can be made in the aim to try and avoid/prevent recruitment problems:

- if financially possible, perform a pilot study to investigate the feasibility of recruitment and examine potential threats before going full scale; and 
- use of dedicated research staff in larger general practices, or involving trained GP assistants in an early stage of the research in smaller practices, might be an effective way to support GPs to outsource eligibility screening and study entry.

For future research, recommended topics are:

- to address important research questions with clear clinical relevance and of daily interest for GPs and their patients.^31^ The protocol and data collection should be straightforward, to have the least disruptive impact on regular care and doctor–patient interaction;^43^ and
-  to have no or restricted impact on usual care delivery during consultations and only register how care is delivered, to minimise the demands made on GPs and their patients.

To conclude, although multiple factors played a role in the unsuccessful recruitment for this trial, it remains unknown which of the determinants played the most important role.

## Trial registration

Dutch Trial Registry, Trials NTR5120.
